# Pre-interventional transesophageal echocardiography as a reliable predictor of residual shunt following patent foramen ovale closure

**DOI:** 10.1007/s00392-025-02713-5

**Published:** 2025-07-24

**Authors:** Tobias Harm, Monika Zdanyte, Andreas Goldschmied, Álvaro Petersen Uribe, Marc Reinert, Juergen Schreieck, Parwez Aidery, Dominik Rath, Tobias Geisler, Meinrad Paul Gawaz, Michal Droppa

**Affiliations:** Department of Cardiology and Angiology, University Hospital Tübingen, Eberhard Karls University Tübingen, Otfried-Müller-Straße 10, Tübingen, 72076 Germany

**Keywords:** Patent foramen ovale, Transcatheter closure, Residual shunt, Peri-device leak, Transesophageal echocardiography, Paradoxical embolism

## Abstract

**Background:**

Closure of a patent foramen ovale (PFO) is an effective strategy in the prevention of recurrent stroke after cryptogenic stroke. Residual shunt (RS) is a common issue following PFO closure and may affect safety and efficacy. Transesophageal echocardiography (TEE) is the key diagnostic tool, but standardized assessment of morphological parameters to prevent RS remains challenging.

**Aims:**

In this study, we investigate the diagnostic value of different anatomical parameters assessed by TEE to predict RS after PFO closure.

**Methods:**

We consecutively enrolled five-hundred and twenty-seven (*n* = 527) patients undergoing PFO closure. We performed pre-interventional TEE, and after PFO closure, we then screened for RS by TEE at 6-month follow-up.

**Results:**

Pre-interventional TEE measures of PFO morphology revealed significant differences in patients with RS in comparison to those with closed PFO. Incidence of RS was significantly more frequent in patients with atrial septum aneurysm (*p* = 0.022) and increasing PFO size (*p* = 0.025). In patients with RS, we found significantly increased length (*p* = 0.005) of septum primum and PFO tunnel (*p* = 0.036) as well as excursion (*p* = 0.005) of septum primum. By training machine learning models on TEE parameters, stratification of PFO morphology resulted in high diagnostic accuracy to predict RS after PFO closure.

**Conclusions:**

Our study elucidates that a baseline characterization of PFO morphology using TEE improves diagnostic precision to identify patients with RS after PFO closure. A standardized approach might thus enhance the efficacy and safety of transcatheter PFO closure. Prediction of complete closure might reduce complications and allow for a more refined patient selection and treatment.

**Graphical Abstract:**

Prediction of peri-device leak in patients PFO and paradoxical embolism undergoing percutaneous PFO closure. Workflow of this study investigating the association of morphological determinants with an increased incidence of residual shunting assessed through transesophageal echocardiography.

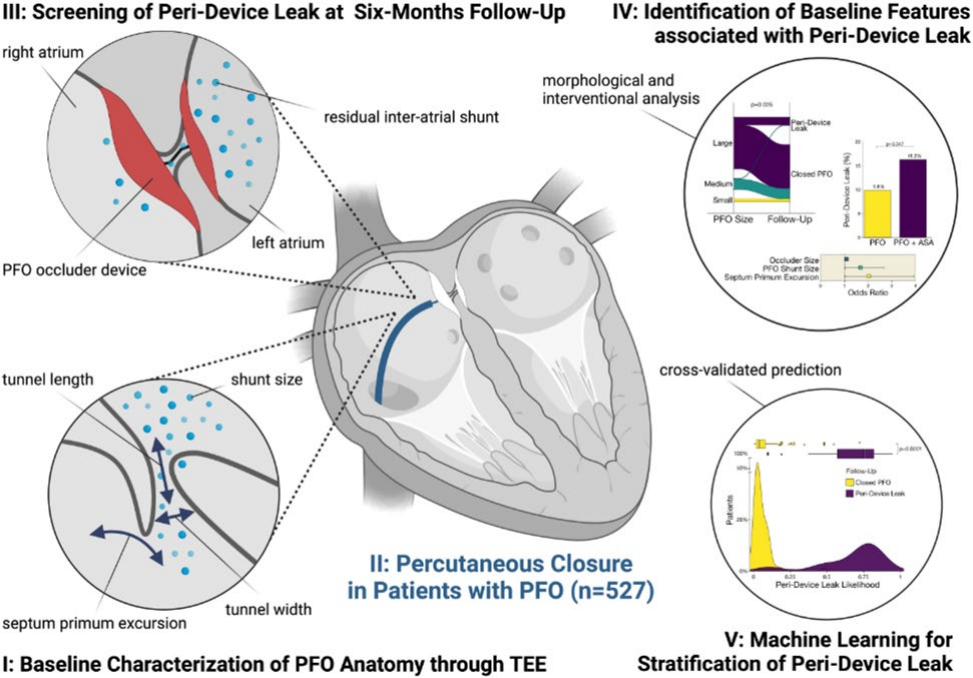

**Supplementary Information:**

The online version contains supplementary material available at 10.1007/s00392-025-02713-5.

## Introduction

Patent foramen ovale (PFO), a congenital inter-atrial shunt, is critically associated with cryptogenic ischemic stroke [1]. The latter refers to stroke of unknown cause after thorough evaluation of further etiologies [2, 3]. The occurrence of PFO-associated stroke is linked to paradoxical embolism passing through the PFO shunt and entering the arterial circulation [1, 4]. After cryptogenic stroke, transcatheter closure of PFO is a safe and effective method to prevent recurrence of paradoxical embolism [3, 5, 6]. Randomized clinical trials (RCTs) demonstrated the superiority of interventional PFO closure in the prevention of stroke when compared to antiplatelet therapy or oral anticoagulants [7–11]. However, RCTs also documented increased rates of atrial fibrillation and procedural complications [11, 12]. Despite improvements in devices and procedural imaging, complete closure of PFO and elimination of large shunts remain challenging. Accurately assessing anatomical landmarks by transesophageal echocardiography (TEE) is a crucial and essential diagnostic step before intervention [3, 13, 14]. One of the most frequent issues is peri-device leak, with incidences reaching up to 25% of patients undergoing PFO closure [11]. The leakage often causes significant residual shunting (RS) and has been identified as an important risk factor for recurrent transitory ischemic attack and ischemic stroke [15–21]. Especially large RS is prone to a higher risk of stroke or TIA recurrence [16]. Repeated embolization and the occurrence of cryptogenic stroke have dramatic consequences, ranging from severe symptoms necessitating hospitalization to adverse events, including death [22, 23]. The morphological features of PFO in patients with RS have not been consistently studied in larger cohorts. Therefore, our objective is to identify the morphological features of PFO by pre-interventional TEE that are associated with the incidence of RS after PFO closure.

## Methods

### Study population

Patients with PFO and paradoxical embolism who underwent interventional PFO closure at our center were enrolled in this retrospective study. Consecutive patients with cryptogenic stroke, TIA, or peripheral arterial embolism attributable to PFO were retrospectively recruited between August 2010 and February 2021 at University Hospital in Tübingen. The study was approved by the local ethics committee (068/2021BO2). The study report followed the STROBE (Strengthening the Reporting of Observational Studies in Epidemiology) guidelines, and the experiments were performed in accordance with the highest ethical standards as laid down in the Declaration of Helsinki.

### Transesophageal echocardiography

All patients underwent pre-interventional TEE using midoesophageal bicaval view (BC) and short axis view (SAX) to assess the baseline morphological characteristics of the PFO anatomy. The following parameters were evaluated: tunnel length, tunnel width, septum primum length, excursion of the septum primum into the right and left atrium, septum secundum thickness, distance from the PFO to the aorta and superior vena cava, and semiquantitative shunt grade (Supplemental Figs. S1–S7). Six months after PFO closure, contrast TEE was repeated to define any residual shunt caused by peri-device leakage. In patients with residual shunt, an additional contrast TEE was repeated after a median follow-up period of 12 months.

### Patent foramen ovale closure

Percutaneous fluoroscopy guided PFO closure was performed by trained experts in interventional cardiology using approved closure devices (Supplemental Table S1) under local anesthesia. A detailed description of the study’s enrolment, echocardiographic workup, procedure, and analysis is available in the supplemental material.

### Statistical analysis

Normally distributed data are represented as mean with 95% confidence interval (CI) and non-normally distributed variables are shown as median with interquartile range (IQR) and were computed with Mann–Whitney *U* test. Categorical data are given as numbers with percentages. Correlation data is based on Pearson’s product-moment correlation coefficient and Spearman’s rank correlation coefficient as indicated. For the final machine learning model, we performed extreme gradient boosting (XGBoost) with tenfold cross-validation and regularization techniques were applied as described previously [24]. A detailed description of statistical tests is provided in the supplemental material.

## Results

### Baseline characteristics

We enrolled a total of 527 patients who underwent PFO closure. Percutaneous PFO closure was successful in all studied patients. The baseline demographic characteristics of the patient cohort are presented in Table 1.

**Table 1:** Baseline characteristics of PFO patient population. Significant values (*p* < 0.05) are highlighted

	All (*n* = 527)	Closed PFO (*n* = 458; 87.1%)	Residual shunt (*n* = 68; 12.9%)	*p*-value
Female, *n* (%)	193 (36.6)	167 (36.4)	26 (38.2)	0.767
Age, years (median, IQR)	55 (46–63)	55 (46–63)	56 (49–61)	0.916
Body mass index (median, IQR)	26.1 (23.9–28.7)	26 (23.8–28.4)	27.7 (25–31.7)	0.201
Indication of PFO closure				
Ischemic stroke, *n* (%)	383 (72.7)	325 (70.8)	58 (85.3	**0.012**
TIA, *n* (%)	85 (16.1)	79 (17.2)	6 (8.8)	0.079
Peripheral arterial embolism, *n* (%)	43 (8.2)	39 (8.5)	4 (5.9)	0.462
Other, *n* (%)	18 (3.9)	18 (3.4)	0 (0)	0.097
Cardiovascular risk factors				
Arterial hypertension, *n* (%)	165 (31.3)	146 (31.8)	19 (27.9)	0.521
Hyperlipidemia, *n* (%)	364 (69.1)	308 (67.1)	56 (82.4)	**0.011**
Diabetes mellitus, *n* (%)	31 (5.9)	24 (5.2)	7 (10.3)	0.098
Smoking, *n* (%)	56 (10.6)	47 (10.2)	9 (13.2)	0.454
Obesity, *n* (%)	71 (13.5)	56 (12.2)	15 (22.1)	**0.026**
RoPE score (median, IQR)	5 (4–6)	5 (4–6)	5 (5–5)	0.563
Medication on admission				
Aspirin, *n* (%)	98 (18.6)	87 (19)	11 (16.2)	0.577
P2Y12 inhibitors, *n* (%)	21 (4)	20 (4.4)	1 (1.5)	0.256
LMWH, *n* (%)	51 (9.7)	49 (10.7)	2 (2.9)	**0.044**
DOAC, *n* (%)	366 (69.5)	313 (68.2)	53 (77.9)	0.103
6 months follow-up				
Death	0 (0)	0 (0)	0 (0)	-
TIA/stroke	6 (1.1)	4 (0.9)	2 (2.9)	0.133
Peripheral embolism	2 (0.4)	2 (0.4)	0 (0)	0.586
Myocardial infarction	0 (0)	0 (0)	0 (0)	-
TIMI major bleeding	0 (0)	0 (0)	0 (0)	-
TIMI minor bleeding	2 (0.4)	1 (1.4)	1 (0.2)	0.117

*DOAC* direct oral anticoagulants, *IQR* interquartile range, *LMWH* low molecular weight heparin, *RoPE* risk of paradoxical embolism, *TIA* transient ischemic attack, *TIMI* thrombolysis in myocardial infarction

PFO shunt size was predominantly large (81.2%), atrial septum aneurysm (ASA) was present in 194 (36.8%) patients, and 22.8% of the patients (*n* = 120) received a device with RA disk size above 25 mm. After a median follow-up period of 6 months, 68 individuals (12.9%) showed peri-device leak with right-to-left interatrial shunting. After 12 months follow-up, closure rate increased to 94.4% (Supplemental Table S2).

Among patients with RS, the primary indication for PFO closure was significantly (*p* = 0.012) more frequent ischemic stroke (85.3%) compared to those with complete PFO closure (70.8%) (Table 1).

### Echocardiographic predictors of residual shunt

**Fig. 1:**
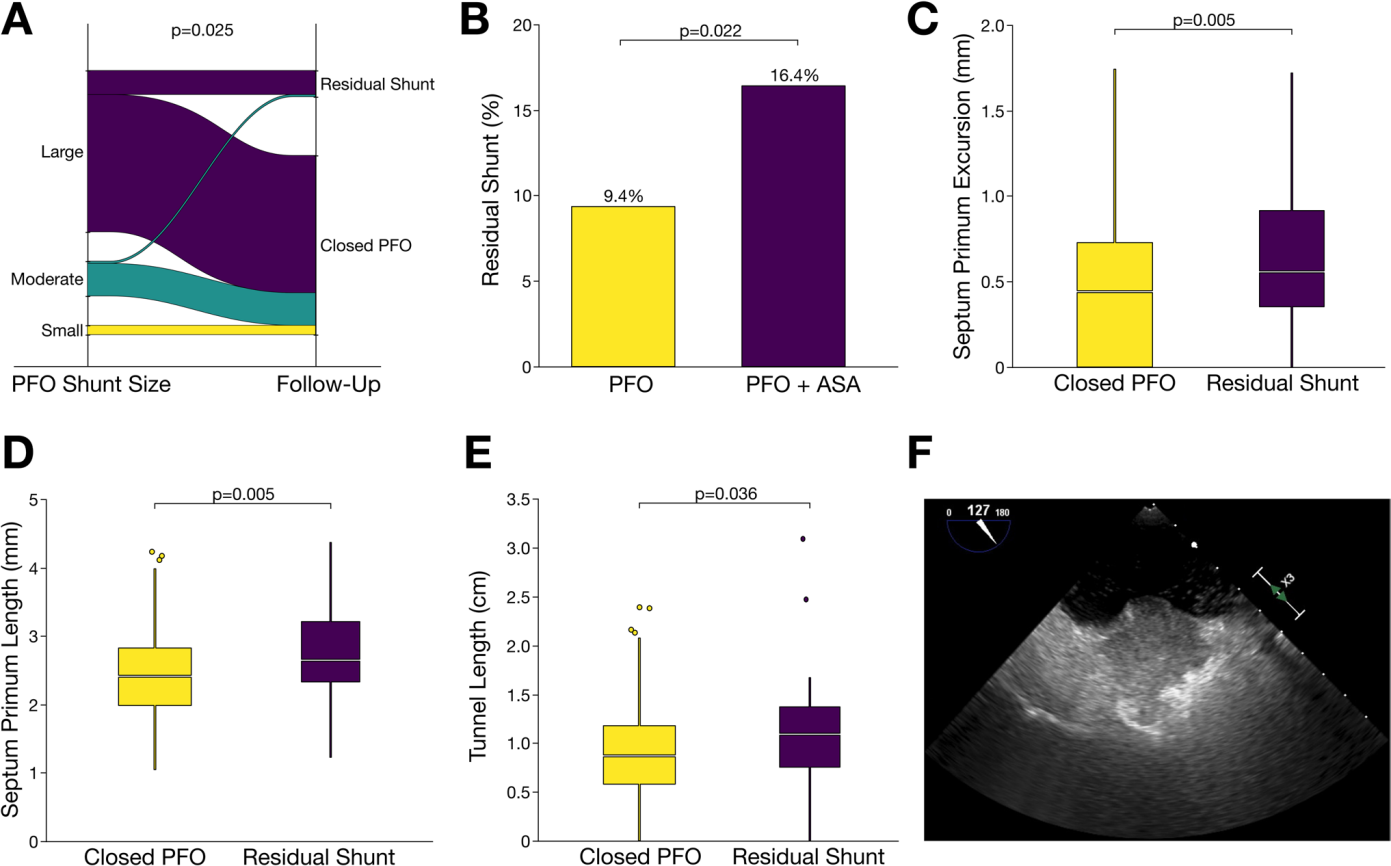
Morphological characterization of PFO through transesophageal echocardiography in patients with paradoxical embolism undergoing percutaneous PFO closure. **A** Sankey plot illustrating the association of residual shunt after 6 months and PFO shunt size at baseline. Persistence of inter-atrial shunt was significantly (*p* < 0.05) higher in case of large or moderate PFO size and did not occur in patients with small shunt size. **B** Proportion of patients with residual shunt (RS) after 6 months was significantly increased (*p* < 0.05) in case of PFO with atrial septum aneurysm (ASA). **C** Box plots demonstrate significantly (*p* < 0.05) increased length of septum primum at baseline in patients with RS. **D** Septum primum length was significantly (*p* < 0.05) increased in patients with RS. **E** Likewise, tunnel length of PFO was significantly (*p* < 0.05) increased in patients with RS when compared to those with successful PFO closure. **F** Representative echocardiographic image depicting the anatomical complexity of PFO associated with RS following transcatheter closure

In patients with RS, we found divergent morphological features assessed through pre-interventional TEE. Among those with incomplete PFO closure, shunt size varied significantly (*p* = 0.025). Of the patients with RS, 91.5% exhibited a large PFO shunt, 8.5% had a moderate shunt, and none had a small shunt in the screening TEE (Fig. 1A). Furthermore, residual shunt was associated with ASA (*p* = 0.022) as incomplete closure occurred in 16.4% of patients with ASA compared to 9.4% of those without ASA (Fig. 1B). Subsequently, excursion of the septum primum, contributing to the definition of ASA, was significantly increased (*p* = 0.005) in patients with RS (Fig. 1C). Additionally, the length of the septum primum was significantly greater (*p* = 0.005) in patients with incomplete PFO closure compared to those with complete closure (Fig. 1D). Another important feature related to the anatomical complexity of the PFO was tunnel width, which was significantly increased (*p* = 0.036) in patients with RS in contrast to those with complete PFO closure (Fig. 1E). A comparison of baseline echocardiographic characteristics identified large PFO shunt size, enhanced septum primum excursion, tunnel width, and device size as significant (*p* < 0.05) predictors of residual shunt in univariate regression analysis (Supplemental Table S3). By derivation of cutoff values from ROC analysis of echocardiographic parameters linked with RS, a large PFO in this study was defined by tunnel width >  10 mm, tunnel length > 1.5mm, septum primum excursion >  10 mm (Supplemental Table S3).

Thus, various anatomical patterns were directly associated with RS and thus determine the difficulty of complete transcatheter closure of PFO (Fig. 1F).

### Device-associated risks for incomplete PFO closure

**Fig. 2:**
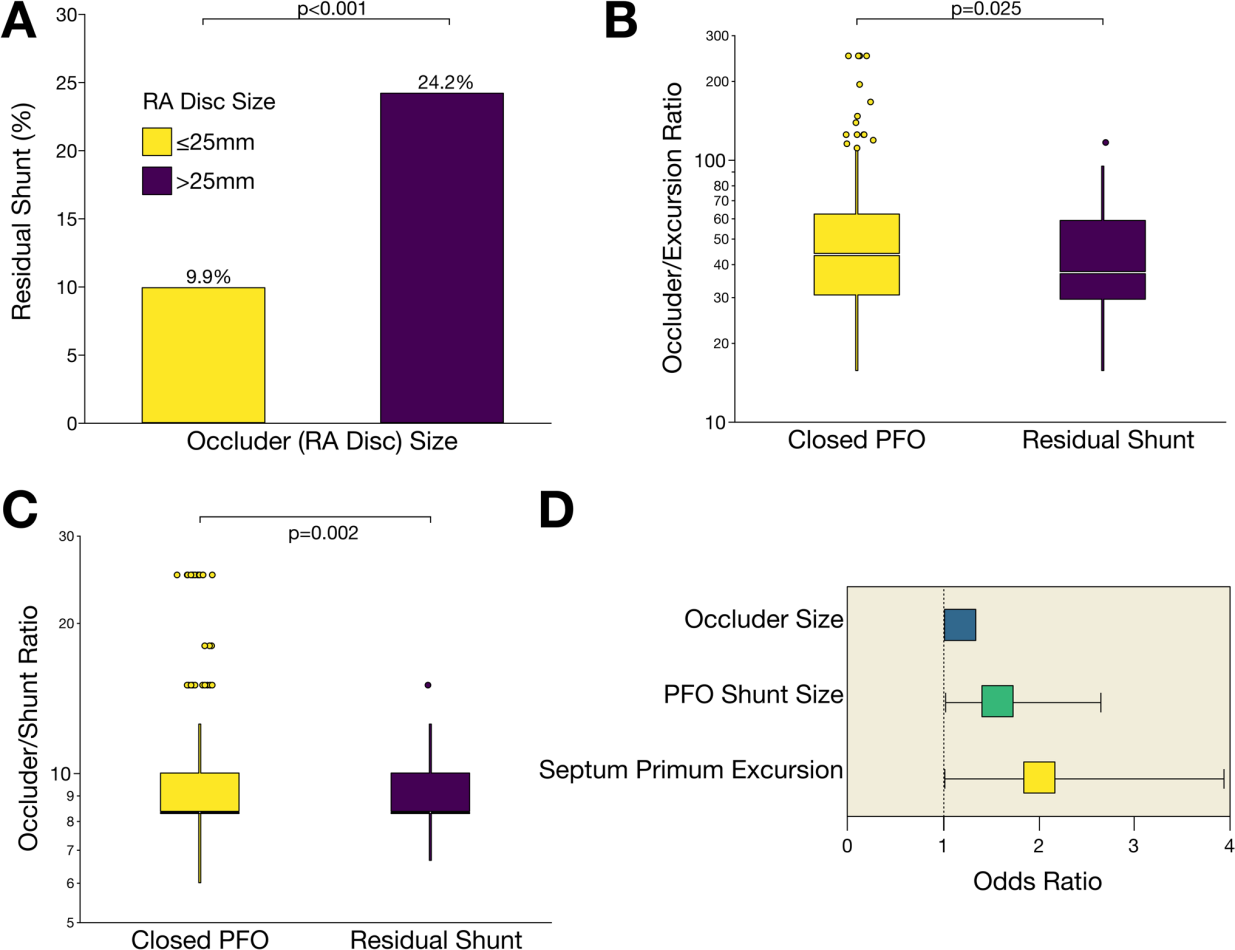
A mismatch between device size and anatomical characteristics is linked to a significant increase in residual shunt (RS) leak following PFO closure. **A** In patients with PFO receiving a closure device with right atrial (RA) disk size diameter greater than 25 mm, the occurrence of peri-device leak was significantly (*p* < 0.05) higher compared to those who received a smaller device. **B** The ratio between RA disk size and excursion of septum primum was significantly (*p* < 0.05) lowered in patients with RS in contrast to those with a successfully closed shunt. **C** Similarly, the ratio between device size and degree of shunt size at baseline was significantly (*p* < 0.05) decreased in patients with RS post PFO closure at six months follow-up. **D** Multivariable regression analysis identified occluder disk size, PFO shunt size, and excursion of septum primum as independent predictors of ineffective PFO closure. Odds ratio of the significant (*p* < 0.05) features is presented as median as well as the lower and upper quartile

In this study, we observed that patients with a larger device size (right atrial disk diameter ≥  30 mm) had a significantly higher incidence of RS compared to those who received a smaller device (24.2% versus 9.9%, *p* < 0.0001) (Fig. 2). The frequency of specific occluder types significantly varied between patients with residual shunt and those with complete closure (Supplemental Table S4). However, when considering morphological characteristics, the ratio between RA disk size and septal excursion was significantly lower (*p* = 0.025) in patients with RS compared to those with closed PFO (Fig. 2B). Similarly, the ratio between occluder size and the degree of right-to-left inter-atrial shunt was significantly lower (*p* = 0.002) in patients with RS (Fig. 2C).

Subsequent, multivariable logistic regression analysis, standardized for closure device size, identified PFO shunt size (OR 1.65, 95% confidence interval [CI] 1.02–2.64; *p* = 0.022) and septum primum excursion (OR 1.99, 95% CI 1.01–3.93; *p* = 0.048) as independent predictors of RS, with a high predictive accuracy of the model (area under the curve [AUC] 0.68, 95% CI 0.67–0.69; *p* < 0.0001) (Fig. 2D and Table 2).

**Table 2:** Identification of morphological determinants and device-related features independently associated with residual shunt post PFO closure

Variable	OR	(95% CI)	*p*-value	AUC	(95% CI)
Occluder (RA disk) size	1.06	1.02–1.1	**0.001**	0.680	0.673–0.687
PFO shunt size	1.64	1.02–2.64	**0.022**		
Septum primum excursion	1.99	1.01 primum excursion 3.93	**0.048**		

Multivariable nominal regression model assessing the occurrence of residual shunt after percutaneous PFO closure. *AUC* area under the curve, *95% CI* 95% confidence interval, *OR* odds ratio, *PFO* patent foramen ovale, *RA* right atrium

### Prediction of residual shunt by machine learning

**Fig. 3:**
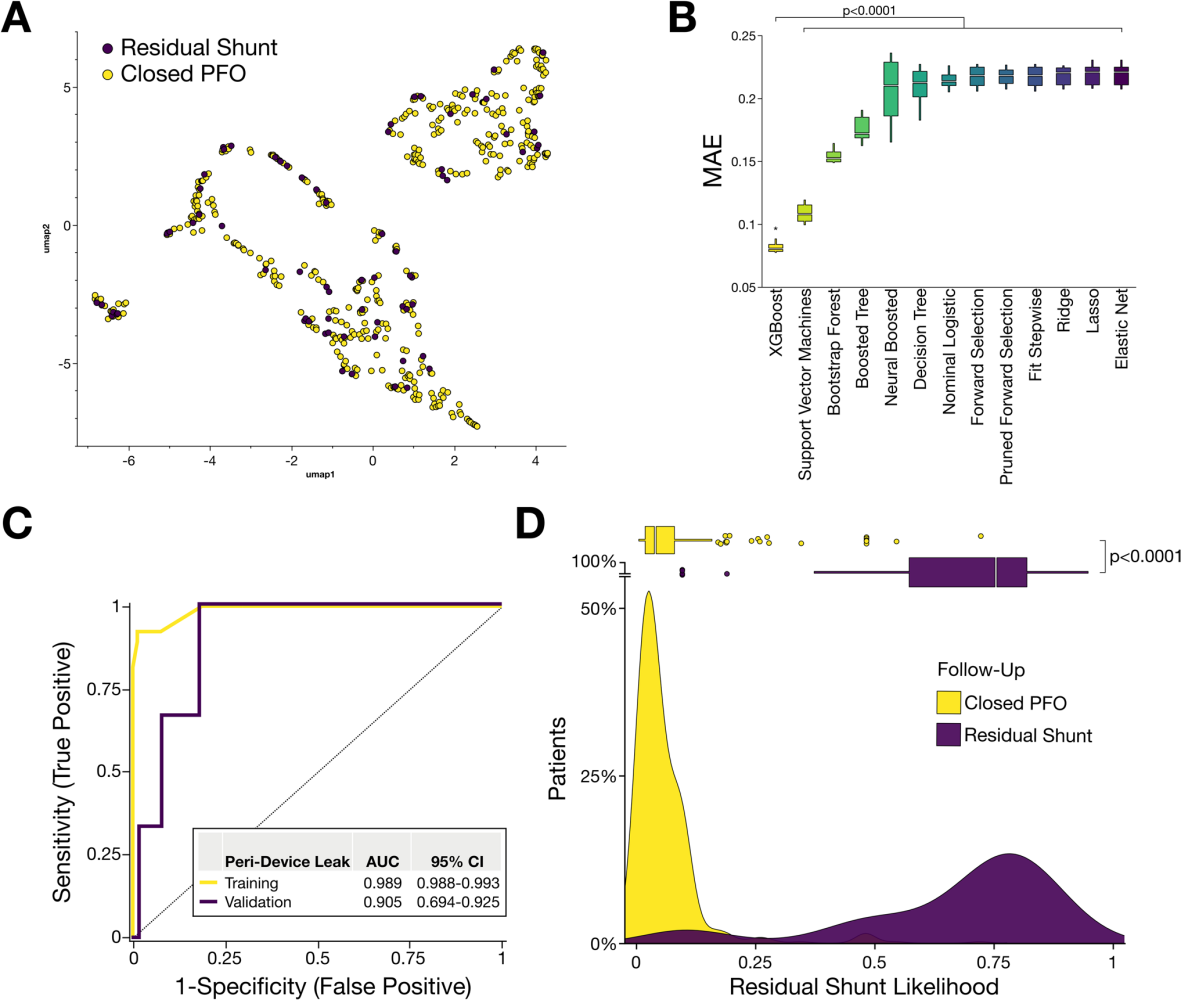
Machine learning analysis of echocardiographic data enhances diagnostic accuracy for predicting residual shunt following percutaneous PFO closure. **A** Unsupervised Uniform Manifold Approximation and Projection (UMAP) of all TEE data of PFO anatomy at baseline unveiled a minor separation between patients with RS and those with successful closure after six months. **B** Comparison of model performance between various machine learning algorithms to predict RS. Extreme gradient boosting (XGBoost) unveiled a superior (*p* < 0.0001) mean absolute error (MAE). **C** Receiver operating characteristic (ROC) analysis of XGBoost algorithm to estimate the likelihood of RS. The supervised cross-validated machine learning model demonstrated a high diagnostic accuracy based on area under the curve (AUC). The model was trained (yellow curve) in a random set of patients and validated (purple curve) using a tenfold cross-validation loop. **D** The transformed likelihood for predicted peri-device leak based on the XGBoost model of baseline echocardiographic data demonstrated a high sensitivity (*p* < 0.0001) in distinguishing between patients with RS (purple) and those with successful closure (yellow) after 6 months

To stratify patients at risk for incomplete PFO closure, we utilized machine learning to integrate all echocardiographic data. Our aim was to determine whether unsupervised analysis of this data would be suitable to reveal clusters of patients at risk for RS. However, uniform manifold approximation and projection (UMAP) analysis for dimension reduction showed a heterogeneous distribution of patients at risk, with no clear segregation into distinct clusters (Fig. 3A). Thus, this finding highlights the importance of supervised machine learning, as unsupervised clustering failed to identify patients at risk for RS. To underline this hypothesis, we compared various machine learning algorithms (Fig. 3B). In the trained models, XGBoost showed superior prediction accuracy as the median absolute error (MAE) of the model was significantly lower (*p* < 0.0001) compared to other models (Fig. 3B). Therefore, XGBoost was utilized to further estimate the occurrence of RS in patients undergoing percutaneous PFO closure. The cross-validated model showed high predictive accuracy for identifying patients at risk for RS in both the training (ROC AUC = 0.99, 95% CI 0.99–99) and test (ROC AUC = 0.91, 95% CI 0.69–0.93) cohorts (Fig. 3C). Additionally, the model achieved a sensitivity of 0.79 and specificity above 0.99 in the overall cohort, reflecting its superior accuracy in identifying patients at risk for RS (Table 3 and Supplemental Fig. S8). Correspondingly, transformed risk scores from the predictive machine learning model significantly (*p* < 0.0001) distinguished between patients with RS and those with complete PFO closure (Fig. 3D). To assess whether RS can still be accurately predicted in a longer-term context, we applied our trained and cross-validated model (based on 6-month follow-up data) to the extended 12-month dataset. The model again demonstrated a high degree of predictive accuracy (ROC AUC = 0.95) in detecting RS based on pre-interventional TEE features (Supplemental Fig. S9).

**Table 3:** Enhanced diagnostic accuracy to predict residual shunt PFO closure utilizing machine learning of important patient- and device-related features

XGBoost model	Performance in overall cohort (*n* = 527)
AUC	0.986
Accuracy	0.970
Sensitivity	0.794
Specificity	0.996
PPV	0.964
NPV	0.987

*AUC* area under the curve, *NPV* negative predictive value, *PPV* positive predictive value, *XGBoost* extreme gradient boosting

**Fig. 4:**
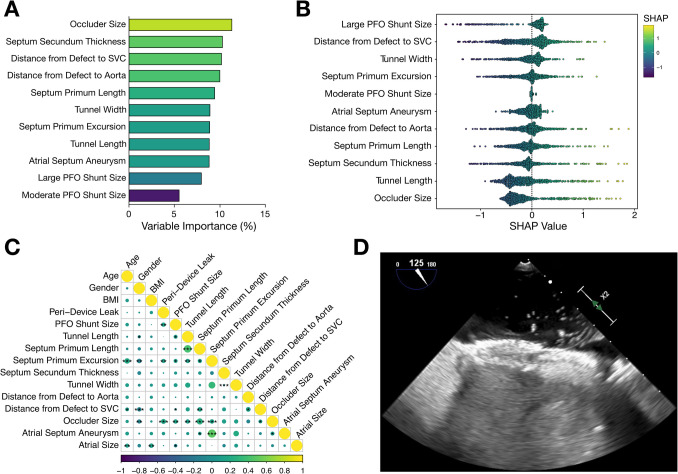
Morphological features of PFO are significantly interrelated and can predict outcomes after percutaneous PFO closure. **A** Most important variables predicting adverse RS based on echocardiographic data at baseline prior to intervention. Included parameters are sorted according to the relative importance of gain parameters and include device- and patient-related features. **B** SHapley Additive exPlanations (SHAP) values for individual features to predict RS. Feature importance is listed in descending order of mean values and colored accordingly, with dots representing patients with PFO. Positive SHAP values indicate an increased likelihood for RS, while negative SHAP values indicate a lower likelihood of the outcome. **C** Comprehensive correlation matrix synopsizing the interrelationship of baseline demographic characteristics, morphological features of PFO, occluder device, and outcome of patients undergoing transcatheter PFO closure. *p < 0.05, **p < 0.01, ***p < 0.001, ****p < 0.0001. **D** Representative TEE image of RS following percutaneous intervention

Incorporation of echocardiographic and device-related features unveiled specific parameters critical for prediction of RS (Fig. 4A). We found that besides morphological features, closure device size was the most important variable contributing to the estimation of RS (Fig. 4A). Furthermore, an in-depth juxtaposition of distinct parameters revealed a divergent relationship between feature values and the outcome variable of RS (Fig. 4B). Particularly, a large PFO shunt size strongly correlated with RS, whereas occluder size was inversely associated with incomplete PFO closure (Fig. 4B). Further, comprehensive correlation analysis unveiled that echocardiographic and occluder-related features are significantly (*p* < 0.05) associated and linked to RS (Fig. 4C). Therefore, consideration of both patient-related and anatomical features, as well as interventional or device-associated variables, is mandatory for complete transcatheter closure of PFO (Fig. 4D).

## Discussion

The key findings of this study are (1) specific anatomical landmarks identified through pre-interventional transesophageal echocardiography are significantly associated with RS following percutaneous closure of PFO and (2) machine learning techniques incorporating baseline morphological features of the PFO can successfully identify distinct patient clusters at risk for RS after PFO closure.

Current antithrombotic treatment regimens aim to reduce the thrombo-ischemic risk in patients with cryptogenic embolic events, albeit at the cost of an increased risk of bleeding [6, 10, 25]. Therefore, transcatheter closure of PFO has been shown to be superior in preventing the recurrence of ischemic stroke by reducing bleeding complications [3, 10]. However, residual interatrial shunting due to significant peri-device leakage may impact the recurrence of adverse ischemic events, including embolic stroke, thereby limiting the efficacy of PFO closure [16]. Thus, precise and careful assessment of anatomical features through TEE is essential prior to intervention [14, 26].

Despite its importance, predictors for RS in patients undergoing PFO closure have not been extensively studied in large cohorts. In the present study, we analyzed TEE characteristics in 527 patients and demonstrated that the incidence of RS was significantly associated with the presence of ASA and large PFO shunt size, tunnel length (≥  10 mm) [27] and tunnel width (>  4 mm) [28]. These findings are associated with an increased stroke risk and are consistent with those reported in previous smaller studies as well as the current definition of complex PFO anatomy [26, 27, 29, 30]. It is tempting to speculate that the excursion and length of septum primum, associated with the presence of ASA, impede stabilization of the defect by PFO closure devices. Moreover, a long-tunnel PFO may cause bunching and distortion of the septal anatomy and potentially contribute to the destabilization of the closure device [27]. It was further notable that patients who received a large device (RA disk ≥  30 mm) had a significantly increased risk for RS. At first glance, this may seem counterintuitive, but it likely reflects the observed association between RS and a larger PFO size with complex anatomy as suggested by previous research [31]. In patients with a larger shunt, longer tunnel, and ASA, a large device is typically selected; yet, it may still fail to completely cover the PFO. Supporting this hypothesis, multivariable analysis revealed that device diameter, shunt size, and the degree of septum primum excursion were independently associated with RS. Among the largest devices, Amplatzer™ and Cardia Ultrasept™ were the only occluders not significantly associated with an increased frequency of residual shunt. On the contrary, the Occlutech™ PFO Occluder was more frequently used in patients with RS and complex PFO anatomy, including long tunnels and ASA, which are themselves strong predictors of RS and may have confounded this observation. Given that this device was the largest available among the manufacturers used in our study, it is tempting to speculate that off-label use of larger devices, such as ASD occluders, or a multi-device strategy involving smaller occluders could potentially enhance closure rates in selected cases. However, the respective subgroups were relatively small, and our study design does not permit definitive conclusions regarding the superiority or inferiority of any specific device manufacturer, particularly in patients with complex PFO anatomy requiring larger occluders. Additionally, besides anatomical complexity, implantation technique and operator experience could have influenced the observed RS rates. Nonetheless, clinicians face challenges in device selection since the device size cannot be increased without restriction. The proximity to other anatomical structures, such as the aortic root or superior vena cava, limits the size of the device. It could be worth considering whether the use of peri-interventional TEE or intra-cardiac echocardiography (ICE) as well as balloon sizing in patients with large PFOs could help to prevent undersizing by ensuring peri-interventional monitoring of the mismatch between device and anatomical features [32, 33]. On the other hand, alternative approaches including transseptal puncture or second transcatheter closure might enhance closure rates [34, 35].

Another important aspect in patients at risk for RS is follow-up. Despite increasing closure rates beyond one year after PFO closure, patients with RS remain at increased risk for recurrent stroke within the first 6 months post-closure [17, 36].

Determination of the duration and intensity of antithrombotic therapy remains challenging, particularly concerning the period beyond the first month post-closure [37]. Patients with morphological features of PFO associated with RS might benefit from an enhanced clinical follow-up, including routine contrast TEE and intensified antithrombotic therapy, to detect RS and subsequently reduce the risk of recurrent embolic events. Conversely, in patients at low risk for RS, shortening the duration of antithrombotic treatment might reduce bleeding complications after PFO closure. In this study, antithrombotic therapy was adapted based on individual ischemic and bleeding risks. In case of RS, DAPT was prolonged up to a year and in case of an enhanced ischemic risk, DOAC therapy was initiated. After persistence of RS beyond 12 months, therapy was generally escalated to DOAC and did not lead to an enhanced bleeding risk. Thus, patients with complex anatomy at baseline might benefit from early escalation of antithrombotic therapy to reduce the thrombo-ischemic risk without incrementally affecting hemostasis. On the other hand, in this study, patients with RS did not show an enhanced thrombo-ischemic risk at both 6- and 12-month follow-up compared to patients with closed PFO. This might be partially reflected by the intensified antithrombotic treatment but further research with a long-term follow-up is mandatory to validate previous findings of an enhanced ischemic risk in patients with persistent RS [16, 25].

In the present study, we demonstrated that machine learning can be effectively applied to predict RS in patients undergoing PFO closure. By training an XGBoost model on echocardiographic data, the algorithm exhibited remarkable diagnostic accuracy in estimating RS risk. With a sensitivity of 79.4% and a specificity of 99.6%, the model might become a powerful tool to rule out patients with low risk for RS while identifying those at increased risk for RS at six months. Furthermore, SHAP analysis revealed that, among other factors, RS was inversely associated with device size. This suggests that selecting a larger device may reduce the risk of RS.

Our study offers several important clinical implications. Firstly, the routine use of pre-interventional TEE allows for precise planning to ensure safe and effective percutaneous PFO closure. Secondly, the data highlight the importance of identifying patients with more complex anatomy (ASA, long tunnel and large shunt) to accurately stratify their individual risk for RS. In these patients, careful device selection, peri-interventional monitoring with TEE or ICE, or alternative implantation techniques such as transseptal puncture may help reduce the risk of persistent RS. Thirdly, patients at high risk for RS, and consequently at an increased risk for recurrent embolic events, require close clinical follow-up, including TEE, and may benefit from intensified antithrombotic treatment.

## Limitations and perspectives

We acknowledge that the robustness of our conclusion, that morphological attributes assessed through TEE can predict RS after PFO closure, is limited by several factors. The occurrence of RS in this study was relatively low, and closure rates might have been influenced by the duration of the clinical follow-up. However, to the best of our knowledge, hitherto, this is the largest study on this topic. Additionally, the absence of established guidelines for selecting closure devices means that patient-specific anatomical characteristics and periprocedural conditions could influence the choice of device size and consequently affect the occurrence of RS. This study reflects real-world decision-making, and the binary outcome of RS supports clinical applicability. However, quantifying RS and extending the follow-up period may improve the detection of clinically significant RS and support risk stratification for associated ischemic events. Standardized guidelines are essential for guiding de-escalation or intensification of antithrombotic therapy in patients undergoing PFO closure, particularly in those with RS. While our study provides valuable real-world evidence on the association between complex PFO anatomy and RS, it is limited by the lack of standardized device selection protocols. Given the current absence of consensus on how anatomical complexity should guide procedural strategies, prospective studies are needed to inform clinical decision-making. Future research, including an ongoing prospective multicenter study, will focus on external validation with the goal of developing a clinically applicable prediction tool for RS. In addition, further research is needed and large-scale, long-term studies with a follow-up beyond one year could enhance the clinical relevance and inform evidence-based patient management. Finally, external validation of the results would increase reliability and enhance the significance of machine learning to predict RS after PFO closure.

## Supplementary Information

Below is the link to the electronic supplementary material.

Supplementary file1 (DOCX 2.91 MB)
