# Supplementary material for: Pre-interventional transesophageal echocardiography as a reliable predictor of residual shunt following patent foramen ovale closure
Source: Clin Res Cardiol. 2025 Jul 24;115(3):484–94. doi: 10.1007/s00392-025-02713-5 (PMC12894160; doi:10.1007/s00392-025-02713-5)
Supplement: Supplementary file 1 — Supplementary file1 (DOCX 2.91 MB) [file 392_2025_2713_MOESM1_ESM.docx]

**Pre-Interventional Transesophageal Echocardiography as a Reliable Predictor of Residual Shunt Following Patent Foramen Ovale Closure**

**Short title:** PFO Closure and Residual Shunt

Tobias Harm MD, Monika Zdanyte MD, Andreas Goldschmied MD, Álvaro Petersen Uribe MD, Marc Reinert, Juergen Schreieck MD, Parwez Aidery MD, Dominik Rath MD, Tobias Geisler MD, Meinrad Paul Gawaz MD, Michal Droppa* MD

Department of Cardiology and Angiology, University Hospital Tübingen, Eberhard Karls University Tübingen, Otfried-Müller-Straße 10, 72076 Tübingen, Germany

**Address for Correspondence:**

Michal Droppa

Department of Cardiology and Angiology

University Hospital Tübingen

Eberhard Karls University Tübingen

Otfried-Müller-Str. 10, 72076 Tübingen, Germany

Tel.: +49 (0) 7071 29 83688

Fax: +49 (0) 7071 29 5749

E-Mail: michal.droppa@med.uni-tuebingen.de

**Supplemental Material and Methods**

**Study population**

Patients with PFO and paradoxical embolism were enrolled in this retrospective study. All patients underwent transesophageal echocardiography prior to catheter-based PFO closure. All patients were treated for cardiovascular diseases and risk factors according to international guidelines at time of study entrance. Consecutive patients with cryptogenic stroke, TIA, or peripheral arterial embolism attributable to PFO were retrospectively recruited between August 2010 and February 2021 at University Hospital in Tübingen. Exclusion criteria of this monocentric study comprised other aetiology of embolism, infective endocarditis or active bacteriemia, intra-cardiac mass, or thrombus at the intended site of implant, as well as poor image quality of transesophageal echocardiography. Paradoxical embolism was considered PFO-related after an extensive and multidisciplinary diagnostic approach to rule out further aetiologies including cardiac embolism and atrial fibrillation, thrombophilia, atherosclerosis, small vessel disease or other vasculopathies. Thus, all patients underwent comprehensive cardiac, neurologic, hematologic, and radiological investigation. Specifically, eligible patients underwent various diagnostic procedures including Holter or prolonged outpatient cardiac monitoring, computed tomography (CT) or magnetic resonance imaging (MRI) for embolism, vascular Doppler imaging, and hypercoagulability work-up. Subsequently, indication for PFO closure was established by a multidisciplinary team including vascular neurologists and cardiologists. A thorough and individual discussion of the benefits and risks of PFO closure considered the attributable likelihood of the index event to the PFO, the risk of recurrent thromboembolic events, the patient's tolerance to anticoagulants, and the patient's lifestyle and family history.

**Patent foramen ovale closure**

Percutaneous PFO closure was performed under local anaesthesia with only fluoroscopic guidance by trained experts in interventional cardiology. The choice of closure device was based on the anatomical characteristics of the PFO and personal experience of the interventionalist. The devices used are listed in the **Supplemental Table S1**. The size of the closure device was selected to ensure complete coverage of the defect, based on pre-interventional analysis of TEE images. In patients with simple PFO anatomy, characterized by a non-prominent ASA (total tissue excursion <10mm), a short tunnel length (<10mm), and a septum secundum thickness <10mm, a 25 mm PFO occluder was typically used. For patients with ASA and long tunnel, larger occluders were selected. Additional parameters, such as the distance from the PFO to the aorta and superior vena cava, were also considered in device selection according to the manufacturer’s instructions. No routine balloon sizing or intracardiac echocardiography was performed during the closure procedure. Patients received either antiplatelet therapy or anticoagulation (direct oral anticoagulants (DOAC) or low molecular weight heparin (LMWH)) until PFO-closure. At discharge, treatment regimens were switched to dual antiplatelet therapy (DAPT) with aspirin and clopidogrel over a period of six months. Aspirin therapy was continued for minimum of 12 month. In patients with deep vein thrombosis or pulmonary embolism, clopidogrel was added to anticoagulation for 6 months after PFO-closure.

**Transesophageal echocardiography**

All Patients underwent pre-interventional TEE using a Philips X8-2 transducer (Philips Medical Systems, Hamburg, Germany) (1–5 MHz) or a Philips EPIQ 7 (Philips Medical Systems) ultrasound system. All echocardiographic examinations and data acquisition were performed by experienced echocardiographers trained in interventional echocardiography using midoesophageal bicaval view (BC) and short axis view (SAX). The comprehensive dataset was further analysed offline using Picture Archiving and Communication System (PACS) of our clinic according to standardized protocols.

Six months after PFO closure, patients attended our outpatient clinic for follow-up contrast TEE to evaluate any residual shunt caused by peri-device leakage. Therefore, agitated saline injection was repeated and quantified according to maximum appearance of bubbles in the LA. For patients with incomplete follow-up of less than six months, echocardiographic results from their most recent follow-up visit, occurring at least 6 weeks after PFO closure, were utilized. All echocardiographic data including the index echo were interpreted in a blinded manner by cardiologists specialized in echocardiography as follows:

**Right-to-left interatrial shunt**

A right-to-left shunt was assessed in the BC or SAX view after intravenous injection of agitated Gelafundin^®^ contrast. The shunt was measured following the Valsalva manoeuvre, within three cardiac cycles after opacification of the right atrium. Semiquantitative grading (Grade 1 <10 bubbles; Grade 2 10-30 bubbles; Grade 3 >30 bubbles) was performed based on the bubble count in the frame with the most contrast (**Supplemental Figure S1**).

**Tunnel length**

The tunnel length was measured in the BC and SAX views (**Supplemental Figure S2**).

**Tunnel width**

The tunnel width was measured in the BC and SAX views at rest, without performing Valsalva manoeuvre (**Supplemental Figure S3**).

**Septum primum length**

The length of the septum primum was measured in the BC and SAX views. In the case of a septum aneurysm, the length was measured in multiple steps (**Supplemental Figure S4**).

**Excursion of septum primum**

The excursion of the septum primum was measured in the BC and SAX views. First, a line was drawn between the atrial roof and the septum secundum. The excursion was measured as the perpendicular distance between this line and the most distal part of the septum primum. The measurement of the excursion was performed for both the right and left atria (**Supplemental Figure S5**).

**Septum secundum thickness**

The thickness of the septum secundum was measured in both the BC and SAX views (**Supplemental Figure S6**).

**Distance from the PFO to the aorta and superior vena cava**

The distance from the PFO to the aortic root was measured in SAX view, the distance from the PFO to the superior vena cava was measured in the BC view (**Supplemental Figure S7**).

**Statistical analysis**

For quantitative measurements of PFO anatomy, highest value from BC and SAX were integrated into further analysis. The presence of atrial septal aneurysm (ASA) was defined as excursion of the septal tissue of >10mm from the plane of the atrial septum into the left (LA) or right atrium (RA) or a combined total excursion left and right of 15mm as described previously.[1]

To analyse the impact of device size on RS, patients were grouped based on the diameter of the right atrial disc into those receiving either large (>25 mm) or small (≤25 mm) occlusion devices. To identify a mismatch between device size and PFO anatomy, we assessed the ratio between the RA disc diameter and the excursion of the septum primum, as well as the semiquantitative shunt grade at baseline, respectively.

Patients baseline characteristics and echocardiographic data were analysed using JMP^®^ Pro Version 17 (SAS Institute, Cary, North Carolina, USA) and R (R Foundation for Statistical Computing, Vienna, Austria). Normally distributed data are represented as mean with standard deviation (SD) and were analysed using student´s t-test. Non-normally distributed variables are shown as median with interquartile range (IQR) and were computed with Mann-Whitney U test. Categorial data are given as numbers with percentages and were analysed using chi-squared test. Correlation data is based on Pearson´s product-moment correlation coefficient and Spearman’s rank correlation coefficient as indicated and comprehensive correlation matrix was created with the “corrplot”[2] package in R.

For prediction of RS, we performed machine learning employing trained models with 10-fold cross-validation loop. Therefore, input data were randomly split into training (90%, n=475) and a test data set (10%, n=52) for all models. The input data were filtered to contain only complete measurements, and missing values were imputed by median values of the predictor. The final model was fit on all data. Comparison of trained models (e.g. extreme gradient boosting [XGBoost], support vector machines, bootstrap forest, boosted tree, neural boosted, decision tree, nominal logistic, forward selection, pruned forward selection, fit stepwise, ridge, lasso, and elastic net) was based on comparison of median absolute error (MAE) utilizing Mann-Whitney U test. XGBoost with cross-validation was applied on all data and hyperparameters were autotuned and variables with gain ≥1% as well as all SHAP values (SHapley Additive exPlanations) were depicted in graphic output within the “beeswarm”[3] package in R. Area under the curve (AUC) confidence intervals of XGBoost models were computed with percentile bootstrap analysis. Likelihood of peri-device leak was derived from XGBoost prediction formula. Final graphic output was performed with different software packages including RStudio and JMP.

**Supplemental Figures and Tables**

**
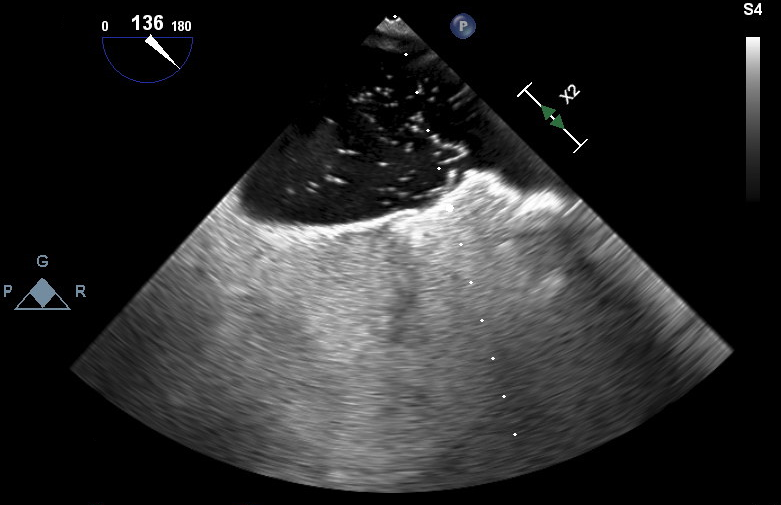
**

**Supplemental Figure S1.** Shunt assessment in the bicaval view after infusion of agitated saline during the Valsalva manoeuvre (grade 3 shunt).

**Supplemental Figure S2.** Measurement of tunnel length in the **(A)** bicaval and **(B)** short axis views.

**Supplemental Figure S3.** Measurement of tunnel width in the **(A)** bicaval and **(B)** short axis views.

**Supplemental Figure S4.** Measurement of the septum primum length in the **(A)** bicaval and **(B)** short axis views. In case of septum concavity, the length was obtained by summing multiple measurements (B1+B2 on figure B).

**Supplemental Figure S5.** Measurement of the excursion of the septum primum in the **(A)** bicaval and **(B)** short axis views. Excursions of the septum were measured on both the left and right atrial side for each patient.

**Supplemental Figure S6.** Measurement fo septum secundum thickness in the **(A)** bicaval and **(B)** short axis views.

**Supplemental Figure S7.** Distance from the patent foramen ovale to **(A)** the aorta in the short axis **(B)** and to the superior vena cava in the bicaval view

**Supplemental Figure S8.** Receiver operating characteristic (ROC) curve depicts the diagnostic accuracy of the XGBoost model to estimate peri-device leakage in the entire study cohort of patients undergoing percutaneous PFO closure. Area under the curve of the validated machine learning algorithm unveiled a good prediction accuracy integrating echocardiographic features at baseline.

**Supplemental Figure S9.** Receiver operating characteristic (ROC) curve illustrating the performance of the XGBoost model in predicting residual shunt at extended 12-months follow-up after percutaneous PFO closure. The area under the curve (AUC) demonstrates strong predictive accuracy of the validated machine learning algorithm based on baseline TEE parameters.

**Supplemental Table S1.** Summary of devices used for transcatheter closure of PFO in patients with paradoxical embolism.

| **Device** | RA Disc  Diameter (mm) | LA Disc  Diameter (mm) | |
| --- | --- | --- | --- |
| **Abbot Amplatzer™ PFO Occluder** | 18 | 18 | |
|  | 25 | 18 | |
|  | 30 | 25 | |
|  | 35 | 25 | |
| Abbott Amplatzer™ Cribriform Septal Occluder | 25 | 25 | |
| Occlutech™ PFO Occluder | 18 | 16 | |
|  | 25 | 23 | |
|  | 30 | 27 | |
| Cardia Ultrasept™ PFO Occluder | 25 | 20 | |
|  | 30 | 25 | |
|  | 35 | 30 | |
| GORE^®^ CARDIOFORM Septal Occluder | 20 | 20 | |
|  | 25 | 25 | |
|  | 30 | 30 | |
|  | | |  |
| *LA, left atrium; PFO, patent foramen ovale; RA, right atrium.* | | |  |

**Supplemental Table S2** Extended one-year follow of patients with residual shunt. 12 individuals from the subgroup of patients with residual shunt at six months (n=68) were lost to follow-up.

| **Follow-up** | **Residual Shunt (n=68)** |
| --- | --- |
| **Transesophageal echocardiography (n=58)** | |
| Residual shunt at 12 months follow-up | 27 (48.2) |
| **Medication (6–12 months after PFO closure) (n=61)** | |
| ASA n (%) | 34 (55.8) |
| Clopidogrel, n (%) | 28 (45.9) |
| Apixaban, n (%) | 8 (13.1) |
| Dabigatran, n (%) | 2 (3.3) |
| Edoxaban, n (%) | 9 (14.8) |
| Rivaroxaban, n (%) | 6 (9.8) |
| **Endpoint (n=60)** | |
| Death | 0 (0) |
| TIA/Stroke | 3 (5) |
| Peripheral embolism | 0 (0) |
| Myocardial infarction | 0 (0) |
| TIMI major bleeding | 0 (0) |
| TIMI minor bleeding | 1 (1.7) |
|  | |
| *ASA, acetylsalicylic acid; DOAC, direct oral anticoagulants; TIA, transient ischemic attack; TIMI, t****hrombolysis in myocardial infarction*** | |

**Supplemental Table S3.** Estimation of residual shunt after PFO closure by individual echocardiographic and device-related features. Univariable nominal regression model assessing factors associated with residual shunt occurrence after percutaneous PFO closure. Cutoff values of echocardiographic and device-related parameters were determined based on the highest Youden index from ROC curve analysis.

| **Variable** | | Cutoff | OR | (95% CI) | p-value |
| --- | --- | --- | --- | --- | --- |
| Distance from Defect to SVC (mm) | | 14 | 1.18 | 0.67-2.08 | 0.556 |
| Distance from Defect to Aorta (mm) | | 15 | 1.05 | 0.47-2.37 | 0.904 |
| Occluder (RA Disc) Size (mm) | | 30 | 3.01 | 1.77-5.51 | **<0.001** |
| PFO Shunt Grade | | 3 | 3.03 | 1.28-7.21 | **0.012** |
| Septum Primum Excursion (mm) | | 10 | 2.50 | 1.28-4.86 | **0.007** |
| Septum Primum Length (mm) | | 23 | 0.95 | 0.76-1.30 | 0.951 |
| Septum Secundum Thickness (mm) | | 10 | 1.89 | 0.43-8.30 | 0.397 |
| Tunnel Length (mm) | | 10 | 1.17 | 0.62-2.20 | 0.626 |
| Tunnel Width (mm) | | 4 | 3.96 | 1.13-13.92 | **0.032** |
|  |  | | | | |
| *95% CI, 95% confidence interval; OR, odds ratio; PFO patent foramen ovale; RA right atrium; SVC, superior vena cava.* | | | | | |

**Supplemental Table S4.** Comparison of used PFO occluder devices between patients residual shunt and patients with successful closure.

| **Device** | | **Patients (%)** | |  |
| --- | --- | --- | --- | --- |
| Occluder | RA/LA Disc  Diameter (mm) | Closed PFO (n=458) | Residual Shunt (n=68) | p-Value |
| **Abbot Amplatzer™ PFO Occluder** | 18/18 | 4 (0.9) | 2 (2.9) | 0.175 |
|  | 25/18 | 240 (52.3) | 22 (32.4) | **0.003** |
|  | 30/25 | 1 (0.2) | 0 (0) | 1.000 |
|  | 35/25 | 6 (1.3) | 2 (2.9) | 0.276 |
|  | Overall |  |  | **0.011** |
| Abbott Amplatzer™ Cribriform Septal Occluder | 25/25 | 4 (0.9) | 1 (1.5) | 0.500 |
| Occlutech™ PFO Occluder | 18/16 | 4 (0.9) | 0 (0) | 1.000 |
|  | 25/23 | 37 (8.1) | 9 (13.2) | 0.167 |
|  | 30/27 | 4 (0.9) | 9 (13.2) | **<0.0001** |
|  | Overall |  |  | **0.001** |
| Cardia Ultrasept™ PFO Occluder | 25/20 | 64 (13.9) | 2 (2.9) | **0.009** |
|  | 30/25 | 56 (12.2) | 10 (14.7) | 0.557 |
|  | 35/30 | 3 (0.7) | 1 (1.5) | 0.426 |
|  | Overall |  |  | 0.165 |
| GORE® CARDIOFORM Septal Occluder | 20/20 | 3 (0.7) | 0 (0) | 1.000 |
|  | 25/25 | 13 (2.8) | 2 (2.9) | 1.000 |
|  | 30/30 | 20 (4.4) | 8 (11.8) | **0.019** |
|  | Overall |  |  | 0.081 |
|  | | | | |
| *LA, left atrium; PFO, patent foramen ovale; RA, right atrium. For subgroup analysis Fisher´s exact test was employed, and significant values are highlighted.* | | | | |

**References**

1. Silvestry FE, Cohen MS, Armsby LB, et al. Guidelines for the Echocardiographic Assessment of Atrial Septal Defect and Patent Foramen Ovale: From the American Society of Echocardiography and Society for Cardiac Angiography and Interventions. *J Am Soc Echocardiogr*. Aug 2015;28(8):910-58. doi:10.1016/j.echo.2015.05.015

2. Taiyun Wei and Viliam Simko (2021). R package 'corrplot': Visualization of a Correlation Matrix (Version 0.92). Available from https://github.com/taiyun/corrplot.

3. Eklund A, Trimble J (2021). _beeswarm: The Bee Swarm Plot, an Alternative to Stripchart_. R package version 0.4.0, <https://CRAN.R-project.org/package=beeswarm>
